# Structural Organization of Mammalian Prions as Probed by Limited Proteolysis

**DOI:** 10.1371/journal.pone.0050111

**Published:** 2012-11-20

**Authors:** Ester Vázquez-Fernández, Jana Alonso, Miguel A. Pastrana, Adriana Ramos, Lothar Stitz, Enric Vidal, Irina Dynin, Benjamin Petsch, Christopher J. Silva, Jesús R. Requena

**Affiliations:** 1 CIMUS Biomedical Research Institute, University of Santiago de Compostela-IDIS, Santiago de Compostela, Spain; 2 Proteomics Unit, IDIS, Santiago de Compostela, Spain; 3 Institute of Immunology, Friedrich Loeffler Institut, Tübingen, Germany; 4 Priocat Laboratory, Centre de Recerca en Sanitat Animal (CReSA), UAB-IRTA, Barcelona, Spain; 5 Western Regional Research Center, USDA, Albany, California, United States of America; 6 Department of Medicine, University of Santiago de Compostela, Santiago de Compostela, Spain; University of Maryland School of Medicine, United States of America

## Abstract

Elucidation of the structure of PrP^Sc^ continues to be one major challenge in prion research. The mechanism of propagation of these infectious agents will not be understood until their structure is solved. Given that high resolution techniques such as NMR or X-ray crystallography cannot be used, a number of lower resolution analytical approaches have been attempted. Thus, limited proteolysis has been successfully used to pinpoint flexible regions within prion multimers (PrP^Sc^). However, the presence of covalently attached sugar antennae and glycosylphosphatidylinositol (GPI) moieties makes mass spectrometry-based analysis impractical. In order to surmount these difficulties we analyzed PrP^Sc^ from transgenic mice expressing prion protein (PrP) lacking the GPI membrane anchor. Such animals produce prions that are devoid of the GPI anchor and sugar antennae, and, thereby, permit the detection and location of flexible, proteinase K (PK) susceptible regions by Western blot and mass spectrometry-based analysis. GPI-less PrP^Sc^ samples were digested with PK. PK-resistant peptides were identified, and found to correspond to molecules cleaved at positions 81, 85, 89, 116, 118, 133, 134, 141, 152, 153, 162, 169 and 179. The first 10 peptides (to position 153), match very well with PK cleavage sites we previously identified in wild type PrP^Sc^. These results reinforce the hypothesis that the structure of PrP^Sc^ consists of a series of highly PK-resistant β-sheet strands connected by short flexible PK-sensitive loops and turns. A sizeable C-terminal stretch of PrP^Sc^ is highly resistant to PK and therefore perhaps also contains β-sheet secondary structure.

## Introduction

Prions are the etiological agents responsible for a diverse set of transmissible fatal neurodegerative diseases of humans and animals, characterized by an abnormal accumulation of prion protein (PrP) [1], [2], primarily in the brain. Prions replicate by converting the normal non-infectious cellular prion protein (PrP^C^) into a prion (PrP^Sc^), via a poorly characterized post-translational conformational transformation. In mice, PrP contains approximately 209 amino acids (numbered 23–231 after cleavage of a 22–mer signal peptide) and has four covalent post-translational modifications: two asparagine N-linked glycans at residues N_180_ and N_196_, a disulfide bridge between residues C_178_–C_213_ and a glycosylphosphatidylinositol (GPI) anchor attached to the C-terminus of the protein (residue S_231_) [2], [3]. Mouse PrP^C^ is a monomer, while PrP^Sc^ is a heterogeneous multimer [2], [3]. There have been no demonstrated covalent differences between mouse PrP^Sc^ and PrP^C^. The only difference between PrP^Sc^ and PrP^C^ is conformational; they are isoforms [2].

The structure of folded, monomeric, recombinant PrP, highly likely to be identical to that of PrP^C^, has been solved by NMR spectroscopy [4] and X-ray crystallography [5]. In contrast, the structure of PrP^Sc^ remains unclear because the insolubility of PrP^Sc^ and the failure to crystallize the heterogeneous PrP^Sc^ multimers prevent the application of the mentioned high resolution analytical techniques. However, a variety of lower resolution instrumental techniques have provided some information about the structure of PrP^Sc^. Unlike PrP^C^, PrP^Sc^ is partially resistant to proteinase K (PK) digestion [2], [6]. The secondary structure of PrP^C^ is largely composed of unstructured and α-helical regions, while PrP^Sc^ is largely composed of β-sheet with little, if any, α-helix [7], [8], [9]. The structure of PrP^Sc^ has also been studied using electron microscopy-based analysis of two-dimensional crystals of the PK resistant core of Syrian hamster (SHa) PrP^Sc^ (PrP27–30) [10], [11] and mass spectrometry(MS)-based analysis of hydrogen/deuterium exchange [9]. Although theoretical models for PrP^Sc^ have been proposed [10], [12], there is an insufficient amount of experimental data to reach a definitive consensus.

In a previous study, we used limited proteolysis to elucidate structural features of PrP^Sc^ [13]. Conformational parameters such as surface exposure of amino acids, flexibility, and local interactions correlate well with limited proteolysis. Peptide bonds located within β-strands are resistant to proteolytic cleavage, whereas peptide bonds within loops and, more rarely, α-helices may be cleaved [14]. Therefore, the targets for limited proteolysis are locally unfolded or highly flexible segments [14]. In our previous study [13], we demonstrated the usefulness of combining limited proteolysis and mass spectrometry (MS) to obtain structural information about two strains of hamster PrP^Sc^. We concluded that the amino-terminal half of PrP^Sc^ features a series of short PK-resistant stretches, presumably β-strands, interspersed with short PK-sensitive stretches, likely loops and turns. Unfortunately, the structural information was largely limited to the N-terminal portion of the protein, as a consequence of the covalent attachment of the heterogeneous GPI anchor and the heterogeneous asparagine-linked sugar antennae to amino acids in the C-terminal portion of the molecule, which prevented MS-based analysis of this part of the molecule.

Here we extended our studies of the structure of PrP^Sc^, by using transgenic (tg) mice expressing PrP^C^ lacking the GPI anchor (GPI^−^) [15]. The GPI^−^ PrP^Sc^ produced by these mice is fully infectious, lacks the GPI anchor, and is largely unglycosylated, which reduces the heterogeneity in the C-terminal portion of the molecule [15], [16]. These properties make it ideal to carry out structural studies, and have allowed us to obtain, for the first time, a complete survey of the whole PrP^Sc^ sequence, regarding its susceptibility to proteolysis.

## Results

### Accumulation of PrP^Sc^ in GPI-anchorless Mice

**Figure 1:**
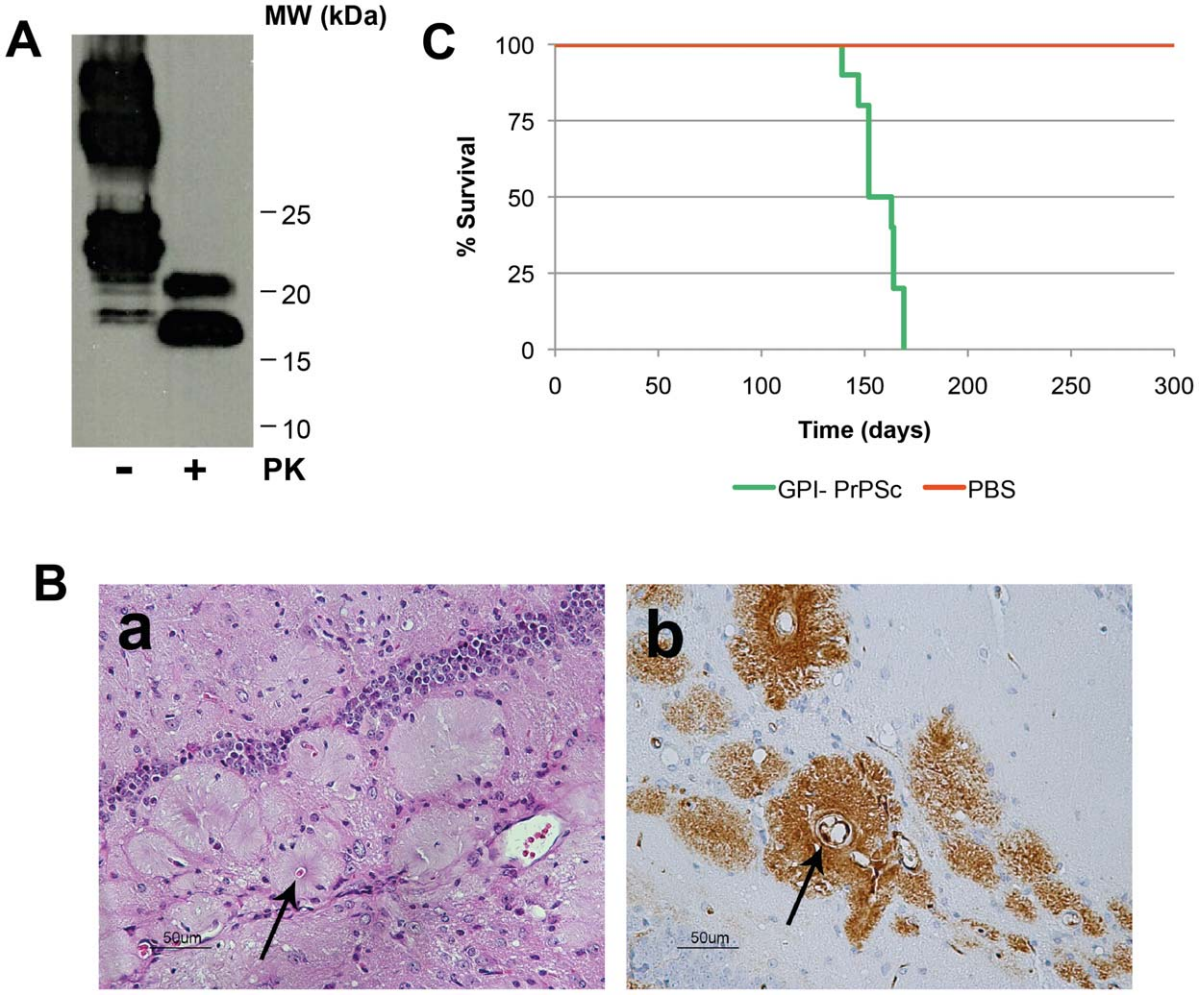
Characterization of GPI^-^ PrP^Sc^. A. Western blot of brain homogenate from scrapie-infected GPI^−^ tg mouse before and after digestion with PK (25 µg/ml); WB probed with SAF83 antibody. **B.** Histopathological and immunohistochemical analyses of scrapie-infected GPI^−^ tg mouse brain. (a) Haematoxylin-eosin staining of the hippocampal formation. (b) IHC staining (antibody 6H4) of the hippocampal formation. **C.** Kaplan-Meier survival curves of wild-type mice (C57BL/6) inoculated with 2% of brain homogenate from scrapie-infected GPI^−^ PrP^Sc^ (green line) and a negative control inoculated with PBS (red line).

Homozygous GPI-anchorless PrP mice were inoculated at 6 weeks of age with the RML strain of murine-adapted scrapie. Three-hundred sixty-five days post-inoculation, the mice were humanely euthanized. Their brains were surgically removed for further biochemical processing. The presence of PrP^Sc^ was confirmed by digesting a portion of some of these brains, after suitable homogenization, with proteinase K (PK) and analyzing the result by Western blot (Figure 1A and S1). The PK treatment yielded the characteristic PK resistant core protein, referred to as PrP27-30 in PK-treated wild-type PrP^Sc^, although in this case its apparent MW is lower, given the lack of GPI and sugars. Histological analysis of brains from several of the infected transgenic mice showed a characteristic PrP accumulation pattern, as previously described [15], [16], with hyaline deposits arranged radially around blood vessels. Those deposits were strongly immunoreactive to PrP monoclonal antibody 6H4. Deposits were also located submeningeally, subventricularly and scattered in the neuropil (Figure 1B). In order to verify that the GPI^-^ PrP^Sc^ was infective, a group of ten wild-type (C57BL/6) mice were inoculated with brain homogenate prepared from one of the infected transgenic mice. All ten of these wild-type mice became ill with clinical signs characteristic of the RML strain of murine-adapted scrapie and were humanely euthanized. The incubation period of the disease was 154±15 days post-inoculation (Figure 1C).

### Identification of PK Cleavage Sites in GPI-anchorless PrP^Sc^ by Mass Spectrometric Detection

We isolated PK-resistant PrP^Sc^ fragments from infected GPI^−^ brains. Purity of this material was assessed by SDS-PAGE followed by Coomassie staining (Figure S2). Using a high resolution Tricine/SDS-PAGE system [17], we compared the distribution of these fragments with that of fragments present in PK-treated unpurified GPI^−^ infected brain homogenate, and found them to be similar, which demonstrates that our purification process isolates all of the PK-resistant fragments (Figure S3). GPI^−^ PrP^Sc^, unlike wild-type PrP^Sc^, permits the use of MS to accurately identify all PK cleavage sites. This allowed us to analyze samples by Western blot (WB) and by MS.

**Figure 2:**
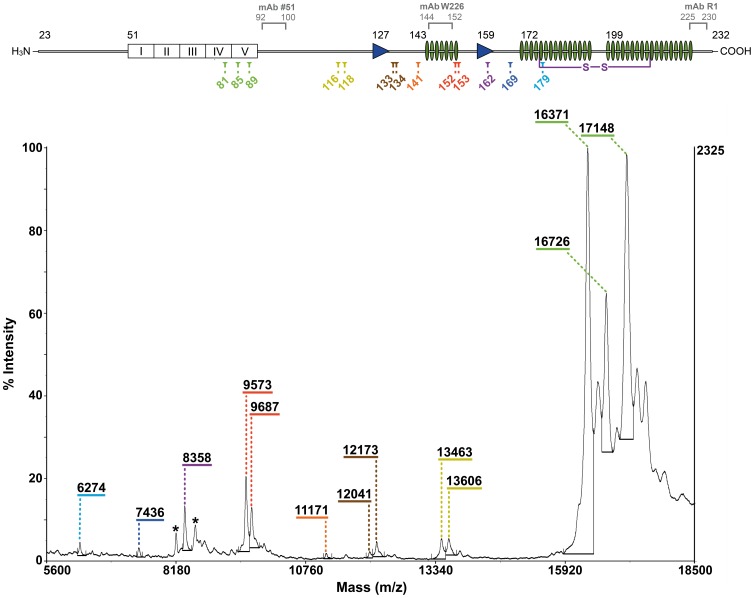
MALDI-TOF spectrum of PK-treated purified GPI ^−^ **PrP^Sc^.** Doubly-charged ions from peptides with m/z 16371 and 17148 are indicated (*). Low resolution in the >16 kDa region precluded identifying unmarked peaks. A scheme of GPI^-^ PrP sequence with PK cleavage points (color coded) and secondary structure of PrP^C^ is included at the top: (octarepeats (□), β-sheets (▸), and α-helices (∥)); epitopes of the mAbs used are also indicated.

We analyzed our samples with high mass accuracy using nano-LC-ESI-Qq-TOF MS (Figure S4) and identified three peaks of 17148, 16728, and 16371 Da (peptides G_81_-S_232_, G_85_-S_232_, and G_89_-S_232_). The smaller peptides were analyzed by MALDI-TOF. MS-based analysis revealed that the seven bands present in the WB (*vide infra*) contained thirteen peptides with MWs of 17148, 16726, 16371, 13606, 13463, 12173, 12041, 11171, 9687, 9573, 8358, 7436 and 6274 Da. By comparing the observed masses with those calculated from the mouse GPI^-^ PrP sequence, we determined that they correspond to peptides G_81_-S_232_, G_85_-S_232_, G_89_-S_232_, A_116_-S_232_, G_118_-S_232_, M_133_-S_232_, S_134_-S_232_, G_141_-S_232_, N_152_-S_232_, M_153_-S_232_, Y_162_-S_232_, S_169_-S_232_ and V_179_-S_232_ (Figure 2 and Table 1). No C-terminally truncated peptides were observed in our MS or WB-based analysis (*vide infra*).

**Table 1:** PK-resistant fragments in GPI^−^ PrP^Sc^.

WESTERN BLOT		MALDI-TOF			
Band	kDa	Peak (Da)	Theoretical mass (Da)	Cleavage point	Peptide
1	17	17148 16726 16371	17148 16729 16371	81 85 89	G_81_ - S_232_ G_85_ - S_232_ G_89_ - S_232_
2	14.6	13606 13463	13605 13463	116 118	A_116_ - S_232_ G_118_ - S_232_
3	13	12173 12041	12172 12041	133 134	M_133_ - S_232_ S_134_ - S_232_
4	12	11171	11172	141	G_141_ - S_232_
5	10.2	9687 9573	9688 9574	152 153	N_152_ - S_232_ M_153_ - S_232_
6	8	8358	8358	162	Y_162_ - S_232_
7	6.7	7436 6274	7436 6278	169 179	S_169_ - S_232_ V_179_ - S_232_

* Entries sharing a color represent PK-resistant peptides of very similar MW that were not resolved on the tricine gel.

### Identification of PK Cleavage Sites in GPI-anchorless PrP^Sc^ by Western Blot

**Figure 3:**
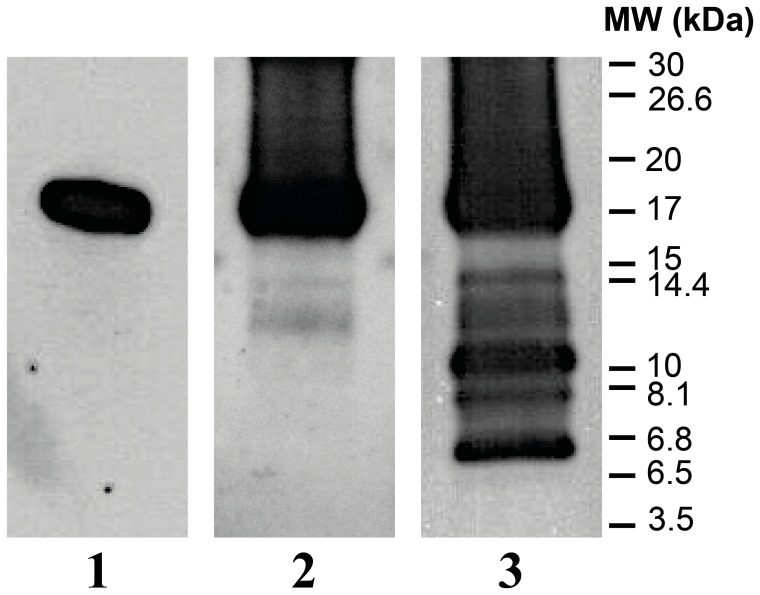
Western blot analysis of PK-resistant GPI ^−^ **PrP^Sc^.** Unpurified GPI^-^ PrP^Sc^ was treated with 25 µg/ml of PK and subsequently deglycosylated with PNGase F. Samples were resolved on Tricine-SDS-PAGE and probed with the monoclonal antibodies, #51 (lane 1), W226 (lane 2), and R1 (lane 3).

In parallel we used Tricine-SDS-PAGE [17] followed by WB to analyze the PK-digested GPI^-^ PrP^Sc^ (Figure 3). When the WB was probed with the antibody #51 (epitope G_92_-K_100_), just one wide band (∼17 kDa) was observed, suggesting a set of cleavage products near G_89_ with no C-terminally truncated fragments. A blot probed with the W226 antibody (epitope W_144_-N_152_), revealed three additional faint bands (∼14.6, 13 and 12 kDa), suggesting three PK cleavage sites between the epitopes of these antibodies. Probing with the C-terminal R1 antibody (epitope Y_225_-S_230_) revealed three more bands (∼10.2, 8 and 6.7 kDa), suggesting three additional cleavage sites near residues Y_149_, P_164_ and V_175_. These bands agree quite well with our MS-based analysis (*vide supra*). In order to exclude the possibility that the observed PK-resistant fragments are the result of the known preference of PK of certain amino acid residues, rather than structural constraints, we subjected a similar amount of freshly refolded, recombinant MoPrP to cleavage by PK. A concentration of PK much lower than that used with mouse GPI^-^ PrP^Sc^, 1 µg/ml, completely destroyed all PrP, leaving no PK-resistant fragments larger than 3.5 kDa (Figure S5). Only PK concentrations below 1 µg/ml yielded some partially resistant fragments, whose sizes do not match those of PK-treated GPI^-^ PrP^Sc^.

### Kinetics of PK Digestion in GPI-anchorless PrP^Sc^

**Figure 4:**
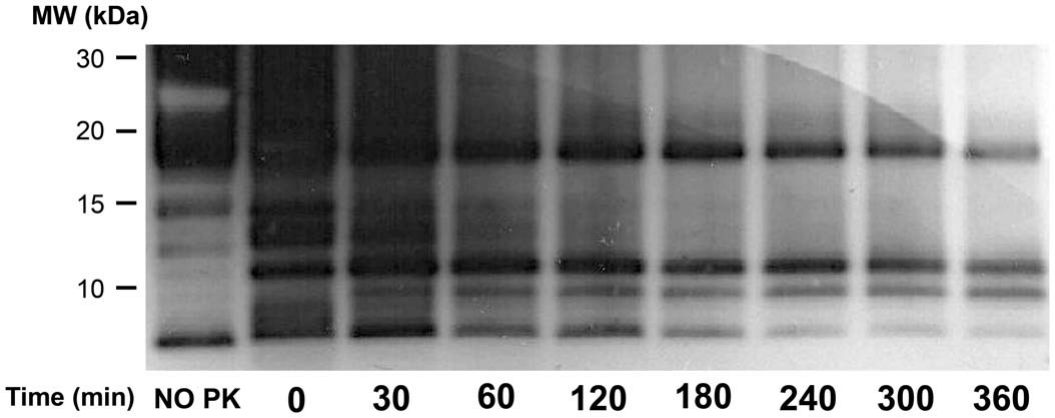
Kinetics of PK digestion of unpurified GPI ^−^ **PrP^Sc^.** Samples were digested with PK (25 µg/ml) and the reaction stopped after 0, 30, 60, 120, 180, 240, 300 and 360 minutes. Samples were treated with PNGase F and subjected to Tricine-SDS-PAGE the blot was probed with R1 antibody.

We performed a PK-digestion time course to determine the relationship of these peptides to one another. A time-dependent reduction in intensity of all PK-resistant bands was observed (Figure 4). The intensities of the 17, 14.6, 13, 12, and 6.7 kDa bands decreased steadily over time. By 240 minutes the intensities of the 17 and 10.2 kDa bands are nearly equal and by 360 minutes the intensity of the 17, 10.2 and 8 kDa bands are similar. These results are consistent with a progressive digestion of GPI^-^ PrP^Sc^ from the N-terminus. This further suggests that different PK-resistant fragments are not from different sub-populations of GPI^-^ PrP^Sc^, instead they are derived from a larger common GPI^-^ PrP^Sc^ peptide.

### PK Cleavage Analysis After Partial Unfolding of GPI-anchorless PrP^Sc^

**Figure 5:**
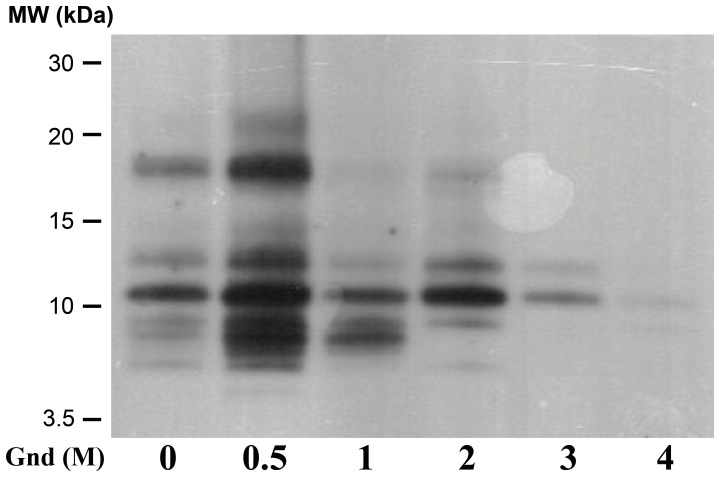
Western blot of PK-digested series of GPI^−^ PrP^Sc^ samples following partial unfolding by guanidine HCl. After guanidine partial unfolding with 0 M, 0.5 M, 1 M, 2 M, 3 M and 4 M and PK treatment (25 µg/ml), the samples were treated with PNGase F and resolved on Tricine-SDS-PAGE. The WB was probed with the R1 antibody.

The above observations were confirmed when the GPI^−^ PrP^Sc^ was partially unfolded with increasing concentrations of guanidine prior to PK cleavage, following the procedure of Kocisko *et al*. [18]. These authors have shown that partial unfolding of PrP^Sc^ with up to 2.5–3 M guanidine is reversible upon dialysis. GPI^-^ PrP^Sc^ became more susceptible to proteolytic digestion in a guanidine-concentration dependent manner. At concentrations above 1 M, the 10.2 and, to a lesser extent, 12 and 8 kDa bands (N_152_-S_232_/M_153_-S_232_, G_141_-S_232_, and Y_162_-S_232_) predominate. Above 3 M guanidine, which renders the unfolding irreversible [18], almost no PK-resistant material remains (Figure 5). These results mirror those of the PK time course (*vide supra*), *i.e.* all of the bands are derived from the progressive N-terminal digestion of a progenitor peptide. In their original report, Kocisko *et al.* identified in SHaPrP^Sc^ partially unfolded with guanidine, a highly stable PK-resistant core starts before position 143 and continues to the C-terminus [18]. Sajnani *et al*. also detected a resistant SHaPrP^Sc^ core starting at position 139/142 [13].

## Discussion

We present a complete survey of susceptibility to limited proteolysis of a PrP^Sc^ strain (Figure S6). The map of PK-susceptible spots: 116–118, 133–134, 141, 152–153, 162, 169, and 179, strongly suggests regions corresponding to loops and turns, while nicks at 81, 85, and 89 signal the frontier between the structured C-terminal and unstructured N-terminal domains of PrP^Sc^. Given the high proportion of β-sheet secondary sctructure derived from FTIR analyses, it is logical to conclude that PK- resistant stretches flanking these spots most likely are strands of β-sheet.

Our results are in excellent agreement with our previous studies of wild-type PrP^Sc^ [13]. Our experiments with two different SHaPrP^Sc^ strains showed the sequence stretches 23–86 (263K), 23–101 (Dy), 117–119, 131–142, and the region around 154 ( =  mouse M_153_) to be sensitive to PK. In the present study, besides confirming these regions as being PK-sensitive, we identified three additional PK cleavage sites in the C-terminal region of GPI^-^ PrP^Sc^ (Y_162_, S_169_ and V_179_).

We did not find evidence of any PK-resistant peptide with an N-terminus beginning beyond V_179_. This is not a consequence of technical limitations, since the Tricine-based SDS-PAGE allows identification of peptides as small as 3.5 kDa (Figure 3). Instead, either this region is completely resistant to PK, or no stable PK-resistant cores remain if PK cleaves beyond that point.

Our results also agree with several studies describing amino-terminally truncated PK-resistant peptides in human CJD PrP^Sc^. Zou *et al.* described human CJD PrP^Sc^ PK-resistant C-terminal peptides spanning from positions 154/156 and 162/167 to the C-terminus [19]. These fragments are analogous to GPI^-^ PrP^Sc^ peptides N_152_-S_232_/M_153_-S_232_ and Y_162_-S_232_, S_169_-S_232_, respectively. Zanusso *et al*. described two additional amino-terminally truncated human CJD PrP^Sc^ peptides (MW of 16/17 kDa) [20], analogous to the GPI^-^ PrP^Sc^ peptides G_141_-S_232_ and M_133_-S_232_/S_134_-S_232_. Kocisko *et al*. used a C-terminal antibody (epitope 217-232) to demonstrate the presence of a number of amino-terminally truncated PK-resistant species in SHaPrP^Sc^ [18]. Using synthetic mouse prions, Bocharova *et al*. identified the regions beginning at 138/141, 152/153, and 162, and extending to the C-terminus as being resistant to PK [21]. This suggests that synthetic prions and PrP^Sc^ share key structural elements, which would explain the capacity of recombinant PrP fibrils to change their conformation, via a “deformed templating” mechanism, to that of PrP^Sc^ [22].

In contrast, relatively few C-terminally truncated peptides have been described. Notari *et al.* reported two human CJD PrP^Sc^ peptides truncated near position 228 [23]. Stahl *et al.* also reported the presence of a peptide truncated at position 228 in PK-treated SHaPrP^Sc^ [24]. The absence of such fragments in our study could be explained by slight differences in sample preparation, or perhaps by the fact that the absence of the GPI-anchor might have an effect on nearby residues.

This conspicuous absence of the C-terminally truncated peptides is a reflection of the stability of the C-terminal region, in GPI^−^ PrP^Sc^ appears to be the most stable part of the molecule, which is inconsistent with the presence of substantial stretches of α-helical secondary structure in that region. Our results agree with Smirnovas *et al*., who showed the C-terminus of GPI^-^ PrP^Sc^ to exhibit extremely low rates of H/D exchange, typical of extensive H-bonding (β-sheet) [9]. These authors showed that an FTIR absorbance band (∼1,660 cm^−1^) previously assigned to α-helical secondary structure in PrP^Sc^ is also present in the spectrum of recombinant PrP amyloid fibrils, which contain no α-helices, and therefore cannot be taken as evidence of the presence of α-helical structure. They concluded that GPI^−^ PrP^Sc^ consists of a series of β-sheet stretches connected by short loops and/or turns, in agreement with our conclusions. Some stretches exhibiting a somewhat higher exchange rate, suggested to overlap with loops/turns, such as 133–148 or 81–118, are consistent with flexible stretches identified in our study, although discrepancies also exist. The limited resolution of both analytical techniques prevents a more exhaustive comparison, but overall both of them agree.

GPI^-^ PrP^Sc^ fibrils are about 3–5 nm wide ([25] and our unpublished results). This constraint means that each PrP^Sc^ monomer must be coiled in such a way as to fit approximately 140–145 residues (∼G_85_–S_232_) into this width. To do so, PrP^Sc^ monomers must necessarily adopt a multi-layer architecture, as seen in SH3 fibers [26] or the HET-s fungal prion domain [27]. The HET-s prion domain packs 70 residues into two β-strands alternating with turns and loops [27]. Wille *et al.* have suggested that PrP^Sc^ fibrils are composed of four rungs of β-strands, based on their interpretation of X-ray diffraction patterns [28]. In this model, each rung would comprise ∼36–37 residues. Positions N_152_-M_153_ lie near the middle of the G_85_-S_232_ sequence, so it is tempting to speculate that they might be located at an exposed position at the border between rungs. This might explain why the N_152_-S_232_ and/or M_153_-S_232_ fragment emerges as the most conspicuous PK-resistant fragment after prolonged treatment with PK or partial unfolding with guanidine (Figures 4 and 5). Positions A_116_-G_118_ might be the border between the two most amino-terminal rungs (approximately G_85_-A_115_ and A_119_-E_151_). On the other hand, our results are partially inconsistent with the location assigned by Govaerts *et al*., using threading algorithms, to residues K_100_-P_104_ and E_145_-R_163_, placed in loops and not rungs [10]. Our data show that the stretches formed by residues K_100_-P_104_, N_142_-E_151_, and Y_154_-Y_161_, are PK-resistant, *i.e*., likely part of a β-strand rung (Figure 2 and Table 1).

In summary, our data support a PrP^Sc^ structure consisting of a series of highly PK-resistant β-sheet strands interspersed with PK-sensitive short flexible loops and turns. Furthermore, the region comprising ∼V_179_ to the C-terminus of PrP^Sc^ is probably composed primarily of β-sheet, as it is highly resistant to PK. Our data are consistent with our previous results (263K and Dy strains) and those of other researchers using SHaPrP^Sc^. Furthermore, they are consistent with those observed for human CJD PrP^Sc^, which suggests that the myriad human, hamster and mouse prions share a common basic structure.

## Materials and Methods

### Ethics Statement

Animal experiments were carried out in accordance with the European Union Council Directive 86/609/EEC. The procedures and animal care were governed by a protocol that was approved by the Institutional Ethics Committee of the University of Santiago de Compostela. All efforts were made to minimize the suffering of the animals.

### Animals

Transgenic heterozygous GPI-anchorless (GPI^-^) PrP mice (tg44(+/−)) were a generous gift from Bruce Chesebro, Rocky Mountain Laboratories, NIH, Montana, USA. Mice were crossed to obtain homozygous GPI^-^ animals (tg44−/−), which were identified by tail DNA analysis using the PCR protocol described by Chesebro *et al*. [15]. Homozygous animals were bred and expression of GPI^-^ PrP confirmed by Western blot (Figure S1). Female mice were intracerebrally inoculated at six weeks of age with 20 µl of a 2% RML-infected mouse brain homogenate (BH), kindly provided by Juan María Torres, CISA, Madrid, Spain. After 365 days post inoculation, the asymptomatic mice [16] were euthanized, their brains surgically removed, rinsed in PBS, and stored at −80°C until needed.

### Preparation of Brain Homogenates and Isolation of GPI-anchorless PrP^Sc^

Mouse BH, 10% w/v, were prepared in PBS, 5% sarkosyl, using a dounce homogenizer (Wheaton Industries Inc, NJ, USA), followed by one pulse of sonication to clarify the homogenate, with an ultrasonic homogenizer probe (Cole Parmer Instrument CO., Chicago IL, USA).

GPI^−^ PrP^Sc^ was isolated using the method of Baron *et al*. [8]. During the purification, total PrP^Sc^ was treated with 10 µg/ml of proteinase K. The final GPI^−^ PrP^Sc^ pellet was resuspended in 100 µl of deionised water or in 20 µl of a 6 M guanidine solution (final concentration 1.75 µg/µl). The stock suspension was stored at 4°C. Its purity was assessed by Coomassie stained SDS-PAGE gel and estimated to be ∼95% pure. The yield of GPI^-^ PrP^Sc^ was ∼35 µg per brain (BCA protein assay).

### Recombinant PrP

Recombinant Mouse PrP(23-231) was expressed in *E. coli*, and purified and refolded in-column on an NTA affinity column (GE Healthcare, Uppsala, Sweden), as previously described [29]. Refolded protein was dialyzed against 10 mM sodium phosphate buffer pH 5.8 and then against d.i. water.

### Limited Proteolysis

Aliquots of BH (10% in PBS, 5% Sarkosyl) were digested with PK (Sigma-Aldrich, St. Louis, MO, USA) in 20 mM Tris-HCl pH 8.5 at 37°C for 1 h unless otherwise stated. Digestion was stopped by addition of Pefabloc (Fluka, Buchs, Switzerland) to a final concentration of 2 mM. Deglycosylation was carried out with 2 µl of PNGase F solution (New England Biolabs, Ipswich, MA, USA) at 37°C for 48 h, according to the manufacturer’s instructions.

### Digestion with PK After Partial Unfolding with Guanidinehcl (Gnd)

Samples of BH (5 µl) were mixed with an equal volume of an appropriate aqueous Gnd solution to yield the desired final Gnd concentration and then incubated at 37°C for 1 h. After incubating, the samples were diluted with buffer (20 mM Tris-HCl pH 8.5) to yield a 0.4 M Gnd solution, which were then treated with PK (25 µg/ml) for 1 h at 37°C. The digestion was stopped by adding Pefabloc (2 mM final concentration) and the protein was precipitated by addition of ice-cold methanol (85% final concentration). The resulting pellets were resuspended in 9 µl of deionized water, and deglycosylated with PNGase F (*vide supra*).

### Tricine-SDS-PAGE and Western Blot Analysis

The precipitated pellets were boiled for 10 minutes in 10 µl of Tricine sample buffer (BioRad, Hercules, CA, USA) containing 2% (v/v) of β-mercaptoethanol. Electrophoresis was performed using precast 10–20% Tris-Tricine/Peptide gels (BioRad, Hercules, CA, USA), in the Criterion System (BioRad, Hercules, CA, USA). The cathode buffer was Tris-Tricine-SDS buffer 1 × (Sigma-Aldrich, St. Louis, MO, USA) and the anode buffer, 1 M Tris-HCl pH 8.9. Electrophoresis was performed at constant voltage (125 volts) for 200 minutes, on ice.

The gels were electroblotted (350 mA, for 150 minutes; 4°C) onto PVDF membranes (Immobilon-P, 0.45 µm; Millipore, Billerica, MA, USA). Membranes were probed with the following monoclonal antibodies: mAb #51 (epitope: G_92_-K_100_), undiluted; W226 (epitope: W_144_-N_152_), at 1∶5000 dilution; or R1 (epitope: Y_225_-S_230_), at a 1∶5000 dilution. Peroxidase-conjugated anti-mouse or anti-human antibodies (GE Healthcare, Little Chalfont, UK) were used as a secondary antibody, as appropriate (1∶5000 dilution). Blots were developed with ECL-plus reagent (GE Healthcare, Little Chalfont, UK). Three sets of partially overlapping MW markers, Peptide Molecular Weight (Sigma-Aldrich, St. Louis, MO, USA), Kaleidoscope Prestained Standard (BioRad, Hercules, CA, USA) and Novex Sharp Protein Standard (Invitrogen, Carlsbad, CA, USA) were run in each analysis to calibrate the MW of the bands.

### Mass Spectrometry

NanoLC/ESI/MS analysis was done with an Applied Biosystems (AB SCIEX, Framingham, MA) model QStar Pulsar equipped with a Proxeon Biosystems (Odense, Denmark) nanoelectrospray source. Samples of the Gnd stock solution (*vide supra*) were loaded automatically onto a C-18 trapping cartridge and chromatographed on a reversed-phase column (Vydac Everest 238EV5.07515, 75 mm × 150 mm) fitted with a coated spray tip (FS360-50-5-CE; New Objective, Inc.). A nanoflow LC system (Dionex, Sunnyvale, CA) with autosampler, column switching device, loading pump, and nanoflow solvent delivery system was used. Elution solvents were A (0.5% acetic acid in water) and B (0.5% acetic acid in 80% acetonitrile/20% water). Samples were eluted at 250 nL/min using a binary gradient (8% B at 0 min to 80% B in a 30 min linear gradient, held at 80% B for 5 min, then back to 8% B for 15 minutes). The QStar Pulsar was externally calibrated daily with human [Glu1]-fibrinopeptide B.

In parallel, 1 µL of the Gnd stock solution was mixed with with 49 µL of sinapinic acid (SA) solution (10 mg/mL SA dissolved in 30% ACN with 0.3% TFA) and analyzed by MALDI-TOF. One half µL aliquots were deposited using the dried-droplet method onto a 384 Opti-TOF MALDI plate (Applied Biosystems, Foster City, CA, USA). MALDI analysis was performed in a 4800 MALDI-TOF/TOF analyzer (Applied Biosystems, Foster City, CA, USA). MS spectra were acquired in linear mode (20 kV source) with a Nd:YAG, (355 nm) laser, and averaging 500 laser shots. The mass of the peptide M_153_-S_232_ (9573 Da) was determined by an iterative calibration approach, using insulin (m/z = 5733), ribonuclease A (m/z = 13682) and lysozyme (m/z = 14305), (Sigma-Aldrich, St. Louis, MO) as internal standards. Then, the signals from the M_153_-S_232_ (9573 Da), G_89_-S_232_ (16371 Da), and G_81_-S_232_ (17148 Da) peptides were used to calibrate the rest of peaks in the spectrum. Masses were matched to PrP fragments with the help of GPMAW 6.0 software (Lighthouse, Odense, Denmark).

### Immunohistochemistry

Immediately after extraction, the brain was fixed in formalin and then sliced into four transversal sections by cutting the brain caudally and rostrally to the midbrain and at the level of the basal nuclei. The sections were dehydrated by equilibration in solutions of progressively higher ethanol concentration and then equilibrated with xylene before being embedded in paraffin. Haematoxylin-eosin was used to stain the 4 µm thick sections. Additional sections were mounted on 3-triethoxysilyl-propylamine-coated glass slides for immunohistochemical (IHC) studies.

These brain sections were deparaffinised, immersed in formic acid containing peroxidase inhibitors, and autoclaved prior to IHC analysis. These autoclaved samples were washed, treated with proteinase K, washed again, and then incubated overnight with the antibody 6H4 (1∶2000, Prionics AG, Schlieren, Switzerland). The sections were developed using the DAKO EnVision system and 3,3′diaminobenzidine as the chromogenic substrate.

## Supporting Information

Figure S1 **Western blot of unpurified GPI** ^−^ **PrP^Sc^ −/+ PK.** Both samples were treated with PNGase F. WB was probed with the #51 antibody.
(TIF)

Figure S2 **Characterization of isolated GPI** ^−^ **PrP^Sc^.** 10 µl of sample were loaded and separated in a 15% gel by SDS-PAGE. The gel was stained by Coomassie blue. The molecular weight of the GPI-less PrP27-30 is ∼16750 Da.
(TIF)

Figure S3 **Western blot of PK-resistant fragments.** In unpurified (1) and purified GPI- PrP^Sc^ (2). Both samples were digested with proteinase K, 25 µg/ml and 10 µg/ml, respectively, treated with PNGase F and resolved on a Tricine-SDS-PAGE gel. WB was probed with the R1 antibody.
(TIF)

Figure S4 **Bayesian protein reconstruction of the nano-LC-ESI-MS spectra of PK-treated purified GPI** ^−^ **PrP^Sc^.** The mass graphs of the three peaks: 17148 Da (top), 16729 Da (middle) and 16371 Da (bottom), identified by ESI-TOF are shown.
(TIF)

Figure S5 **Western blot of recombinant MoPrP(23–231) cleavage by PK.** Samples were digested with different concentrations of PK: 0, 0.2, 1, 5, 10 and 25 µg/ml. Samples were subjected to Tricine-SDS-PAGE and the blot was probed with R1 antibody.
(TIF)

Figure S6 **Schematic representations of the data. A.** A scheme of GPI^−^ PrP sequence, showing the PK-resistant areas (blue squares) and the PK cleavage points and flexible areas (gray line). **B.** Lengthwise comparison of the different peptides found by limited proteolysis and MALDI-TOF analysis (colors match those displayed in Figure 2).
(TIF)
