# Supplementary figures and images for: Structural Organization of Mammalian Prions as Probed by Limited Proteolysis
Source: PLoS One. 2012 Nov 20;7(11):e50111. doi: 10.1371/journal.pone.0050111 (PMC3502352; doi:10.1371/journal.pone.0050111)

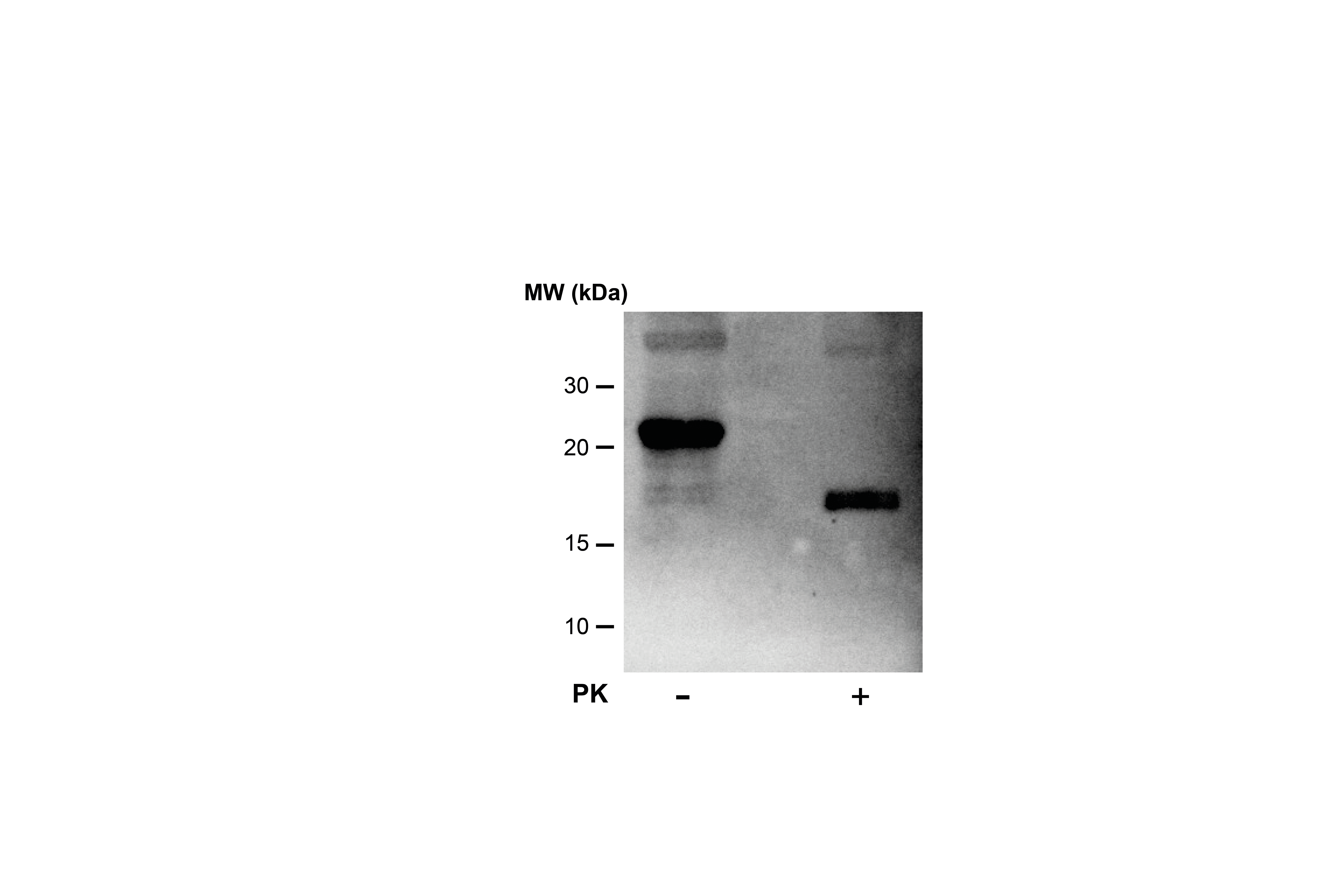

Supplement: Figure S1 — Western blot of unpurified GPI − PrPSc −/+ PK. Both samples were treated with PNGase F. WB was probed with the #51 antibody. (TIF) [file pone.0050111.s001.tif]

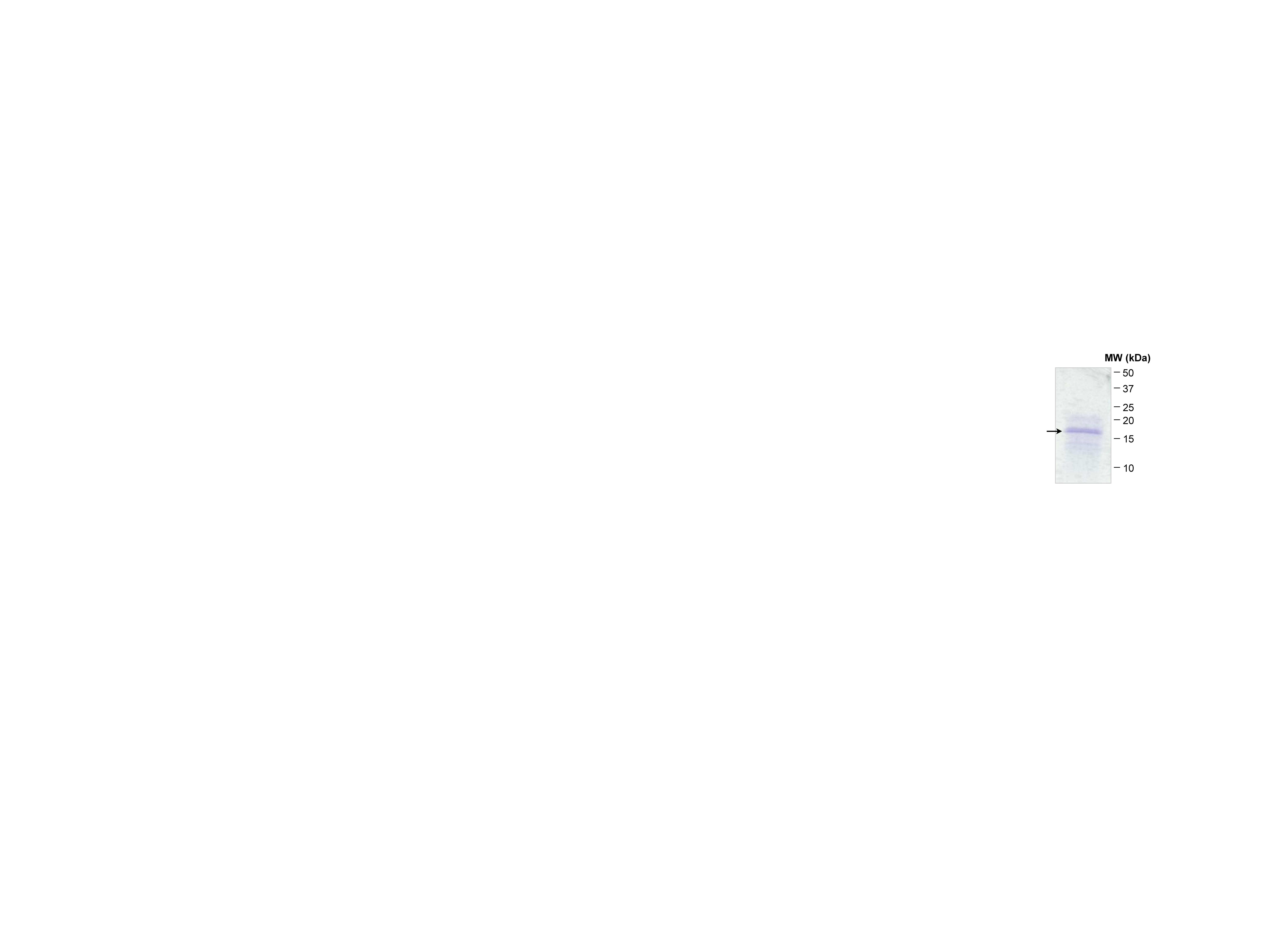

Supplement: Figure S2 — Characterization of isolated GPI − PrPSc. 10 µl of sample were loaded and separated in a 15% gel by SDS-PAGE. The gel was stained by Coomassie blue. The molecular weight of the GPI-less PrP27-30 is ∼16750 Da. (TIF) [file pone.0050111.s002.tif]

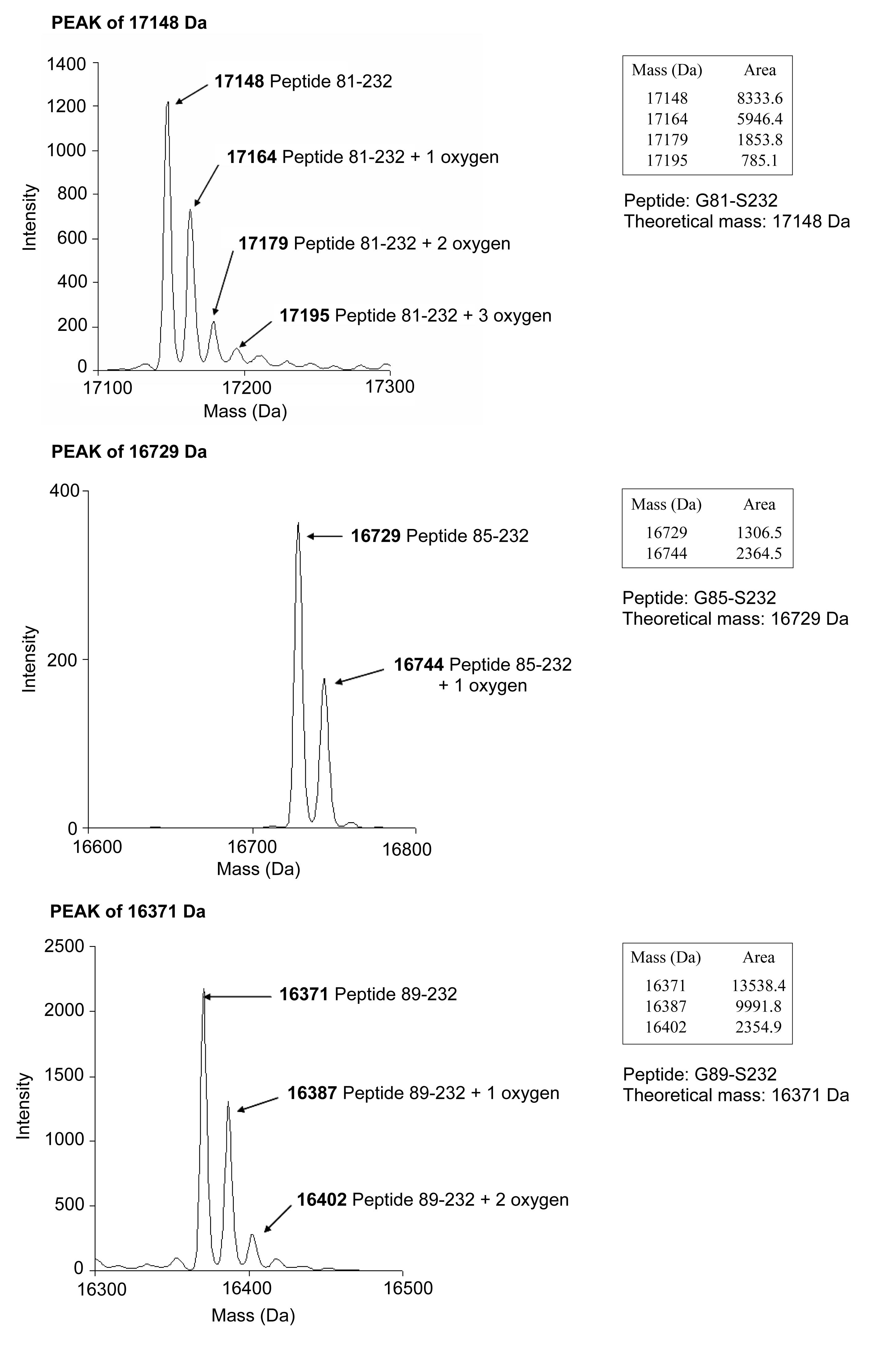

Supplement: Figure S4 — Bayesian protein reconstruction of the nano-LC-ESI-MS spectra of PK-treated purified GPI − PrPSc. The mass graphs of the three peaks: 17148 Da (top), 16729 Da (middle) and 16371 Da (bottom), identified by ESI-TOF are shown. (TIF) [file pone.0050111.s004.tif]

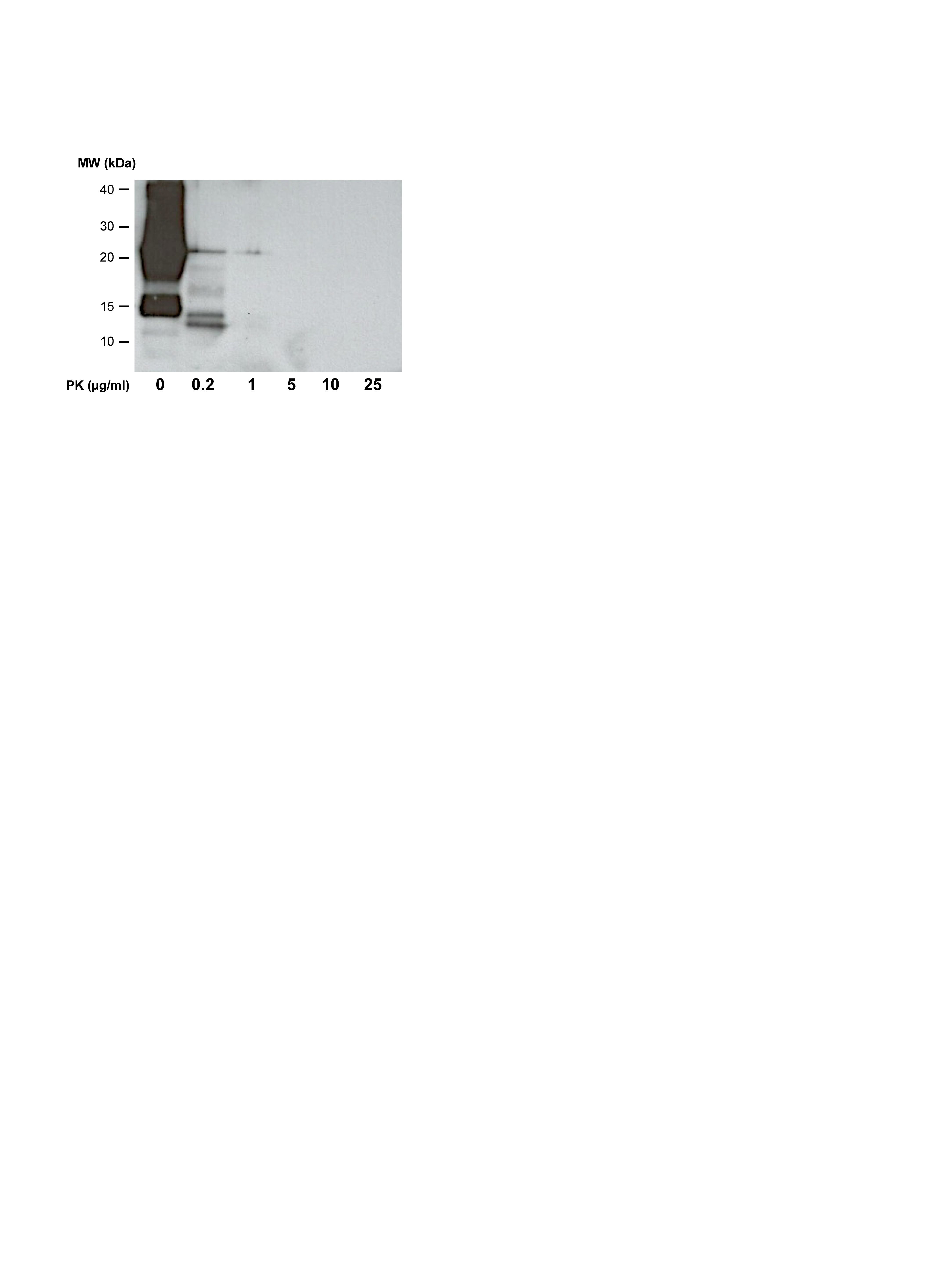

Supplement: Figure S5 — Western blot of recombinant MoPrP(23–231) cleavage by PK. Samples were digested with different concentrations of PK: 0, 0.2, 1, 5, 10 and 25 µg/ml. Samples were subjected to Tricine-SDS-PAGE and the blot was probed with R1 antibody. (TIF) [file pone.0050111.s005.tif]

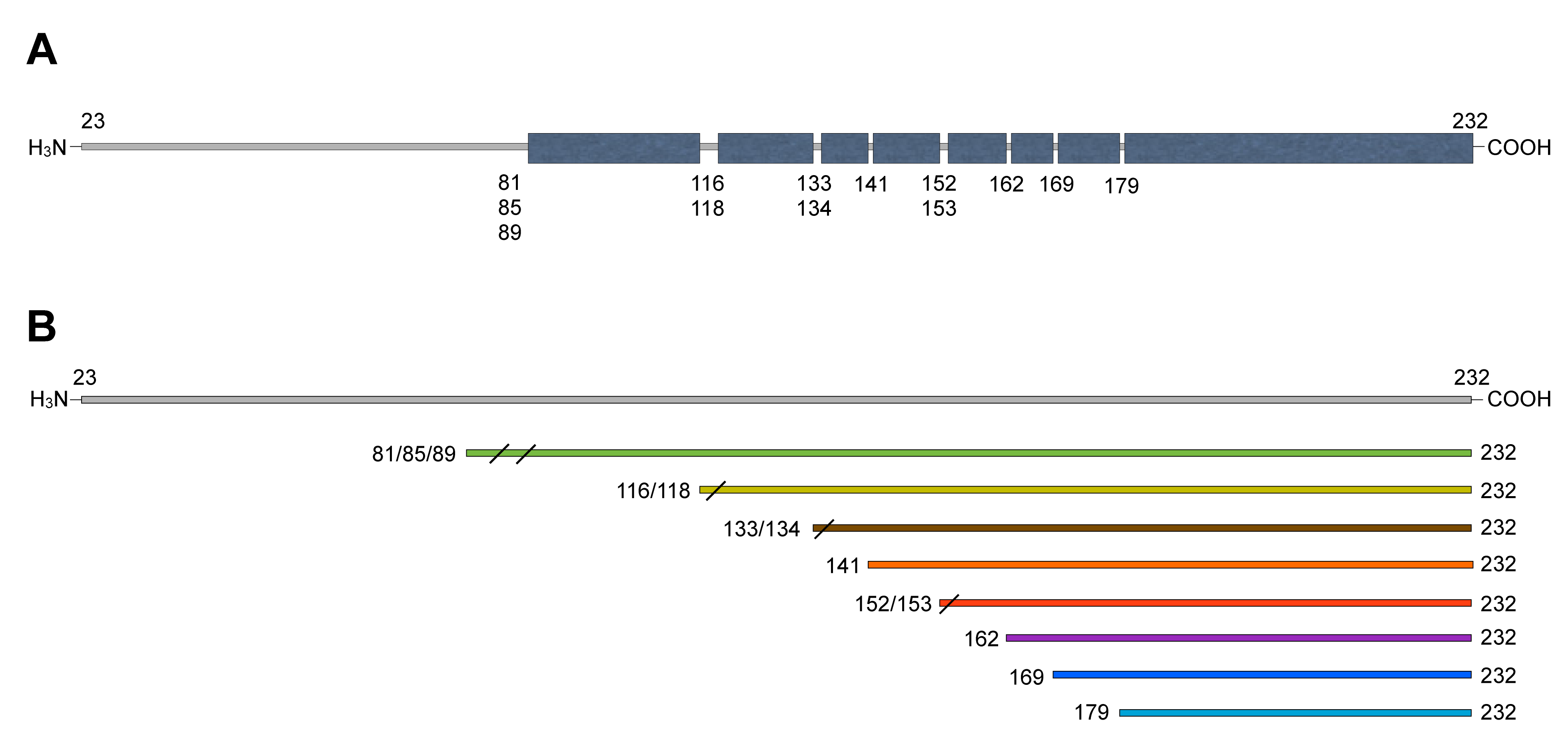

Supplement: Figure S6 — Schematic representations of the data. A. A scheme of GPI− PrP sequence, showing the PK-resistant areas (blue squares) and the PK cleavage points and flexible areas (gray line). B. Lengthwise comparison of the different peptides found by limited proteolysis and MALDI-TOF analysis (colors match those displayed in Figure 2). (TIF) [file pone.0050111.s006.tif]
